# Smartphones in the classroom: power tool or source of distraction? Evidence from a cross-lagged panel study

**DOI:** 10.3389/fpsyg.2026.1831165

**Published:** 2026-09-16

**Authors:** Xiaogang Li, Liping Chen

**Affiliations:** Gansu Forestry Voctech University, Tianshui, Gansu, China

**Keywords:** academic engagement, cross-lagged panel model, digital distraction, smartphone use in education, technology acceptance

## Abstract

**Background:**

The widespread use of mobile devices in educational settings suggests that smartphones may function both as learning tools and as potential sources of distraction. Existing cross-sectional studies have been unable to adequately explain the dynamic causal relationships among technology acceptance, digital distraction, and academic engagement.

**Objective:**

This study aims to examine the reciprocal relationships among these three variables using a longitudinal design, thereby providing empirical evidence for the integration of educational technology.

**Methods:**

A three-wave longitudinal survey (conducted at six-month intervals) was administered to an initial sample of 1,200 high school students from three public secondary schools in eastern China; 1,020 participants (retention rate: 85.0%) were retained through all three waves and included in the final analysis (attrition confirmed as non-systematic via Little’s MCAR test, *p* = 0.78). Data were collected using validated scales assessing technology acceptance, classroom digital distraction, and academic engagement. Cross-lagged panel models (CLPM) and bootstrap mediation analysis (5,000 resamples) were employed to examine reciprocal longitudinal relationships and indirect effects among the variables.

**Results:**

(1) Technology acceptance positively predicted subsequent digital distraction (*β* = 0.12–0.14, *p* < 0.01) but had no significant direct effect on academic engagement. (2) Digital distraction negatively predicted subsequent academic engagement (*β* = −0.23−−0.25, *p* < 0.001). (3) Academic engagement negatively predicted subsequent digital distraction (*β* = −0.08 to −0.09, *p* < 0.05) and positively predicted subsequent technology acceptance (*β* = 0.18 to 0.20, *p* < 0.001). (4) Digital distraction fully mediated the relationship between technology acceptance and academic engagement (indirect effect = −0.032, 95% CI = [−0.059, −0.013]).

**Conclusion:**

The role of mobile phones in education is neither a fixed “power tool” nor an inevitable “distractor,” but rather depends on the dynamic interaction among technological perceptions, behavioral regulation, and learning contexts. The educational value of technology adoption should undergo a transformative process of “assessment,” in which digital distraction serves as a critical gatekeeper. Educational practice should transcend the binary dilemma of “prohibition” versus “overuse” and focus on cultivating students’ self-regulation skills and critical literacy in technology use, thereby advancing an educational philosophy of “teaching according to individual aptitude” in the digital age.

## Introduction

The growing use of mobile technology in learning institutions has drawn a lot of interest among teachers and scholars. The future prospects of smartphones as learning devices do not exclude the threat of becoming distractors that puts the educational practice in a dilemma of continuous existence (Qi, 2019). Whereas the introduction of digital technology into the classroom has become a trend that has been adhered to in many countries globally, empirical evidence indicates that the interaction between mobile phone use in education and academic achievement can not be narrowed down to either a positive or negative relationship (Sweller et al., 2019; Zou et al., 2025). The majority of the available research is based on cross-sectional designs that are also not sufficient in elucidating the nature of dynamic relationships between technology acceptance, digital distraction, and academic engagement and in most cases do not provide insight into the causality of relationships between these variables. To overcome these shortcomings, the proposed research design is based on longitudinal design and cross-lagged modeling in order to investigate the reciprocations of the three factors with an aim of developing empirical findings on the effective implementation of education technology.

### Technology acceptance: a key determinant of the effectiveness of mobile learning

Technology acceptance refers to an individual’s readiness and inclination to use a specific technology and is widely regarded as a core predictor of actual technology use behavior (Davis, 1989; Venkatesh and Thong, 2016). According to the Unified Theory of Acceptance and Use of Technology (UTAUT2; Venkatesh et al., 2012), seven determinants influence technology use intentions: performance expectancy, effort expectancy, social influence, facilitating conditions, hedonic motivation, price value, and habit (Arain et al., 2019).

In educational settings, students’ technology acceptance determines whether they perceive mobile devices as tools for knowledge acquisition and problem-solving or primarily as entertainment platforms. Shahin et al. (2022) found that students who emphasised the educational utility of mobile devices were significantly more likely to engage in knowledge-seeking and academic problem-solving behaviors.

However, technology acceptance does not operate in isolation from its cultural context. Huang and Teo (2020) demonstrated that in East Asian collectivist cultures, students’ classroom technology attitudes are shaped more by social norms and teacher authority than by individual utility judgements. This cultural specificity underscores the importance of examining the relationship between technology acceptance and learning outcomes within the Chinese educational context.

### Digital distraction: the cognitive cost of smartphone use

Digital distraction refers to the redirection of attentional resources away from primary learning tasks toward digital stimuli in educational settings (Salari et al., 2025; Wilson et al., 2010). Unlike conventional distractors, smartphone-induced distraction operates through a dual-processing mechanism: even at minimal usage levels, the mere presence of a mobile device imposes attentional costs by activating anticipatory cognitive monitoring (Levy et al., 2012; Pattnaik and Jena, 2021). Consistent with cognitive load theory (Sweller, 1988), extensive neuroscientific evidence refutes the “myth of multitasking,” demonstrating that parallel cognitive processing is fundamentally constrained by limited working memory capacity (Ophir et al., 2009; Shanmugasundaram and Tamilarasu, 2023). In classroom contexts, phone notifications interrupt focused attention approximately every 90 s, and full recovery of deep concentration may require up to 15–20 min (Liu et al., 2021; note: the original figure of “1,520 min” appears to be a typographical error and should read “15–20 min”). Longitudinal eye-tracking research by Ward et al. (2017) further demonstrated that frequent smartphone checking during complex learning tasks fosters shallow processing cycles that persist over time. Nevertheless, the relationship between digital distraction and learning is not uniformly negative: Khalaf et al. (2022) reported that moderate levels of digital interruption may facilitate creative cognition, suggesting that nuanced, context-sensitive models of digital distraction are warranted.

### Academic engagement: a key mediating variable in learning outcomes

Academic engagement refers to the degree to which students are actively and psychologically invested in their learning activities, comprising three interrelated dimensions: behavioral engagement (participation and persistence), affective engagement (interest and sense of belonging), and cognitive engagement (effortful processing and self-regulation; Bergdahl et al., 2024; Fredricks et al., 2016). As a proximal predictor of academic achievement, engagement occupies a central mediating position between technology adoption and learning outcomes (Ruiz-Alfonso and León, 2016). Research indicates that the relationship between mobile phone use and engagement is non-linear and context-dependent. Cao et al. (2018) demonstrated that low-intensity educational app use enhanced task focus, whereas exceeding a usage threshold was associated with meaningful reductions in academic engagement (a 0.1-unit increase in usage duration corresponded to a 7.3-unit decrease in engagement). Notably, academic engagement may also function as an antecedent of technology acceptance. Longitudinal evidence from Bylieva et al. (2021) showed that highly engaged students selectively adopted technology as a learning tool rather than for passive entertainment, suggesting a reciprocal relationship that necessitates longitudinal investigation. Despite growing interest in this area, research conducted in East Asian educational contexts remains limited, particularly regarding the moderating roles of teacher expectations and peer norms in collectivist settings—a gap the present study aims to address.

### Cross-lagged models: a methodological choice for exploring dynamic relationships among variables

Cross-sectional studies are not able to establish a causal direction between variables, whereas simple longitudinal designs cannot distinguish prevailing changes within an individual and changes between individuals (Hamaker et al., 2015; Zhou et al., 2024). In spite of being the frequently used model to study the relationships of variables over time, the cross-lagged panel model (CLPM) has been accused of through arguing within-individual and between-individual variations (Usami et al., 2019). The random-intercept cross-lagged panel model (RI-CLPM) offers a more accurate causal inference designghaving been used to isolate both the differences between persons and the in-person processes that vary over time (Mulder and Hamaker, 2021). The recent literature indicates that not only some relationships that were viewed as mutually reinforcing might appear as one-way effects only when the between-individual variation is controlled (Núñez-Regueiro et al., 2022). This is one methodological development that has taken place in the research on use of technology. Takahashi et al. (2026) made comparisons of the estimate of the correlation between screen time and academic performance, across various models and concluded that CLPM, though not RI-CLPM supported a two-way every other way prediction between academic performance and future screen time. These results indicate that the intricacy of the experience between the use of technology and learning outcomes might have been simplified through the traditional forms of analysis. Thus, to derive more effective conclusions, this research will use various cross-lagged models to conduct comparative analysis.

Building on prior research, this study integrates perspectives from technology acceptance, cognitive load theory, and academic engagement to explain how mobile phone use functions simultaneously as a learning resource and a source of distraction. Technology acceptance frameworks (e.g., Davis, 1989; Venkatesh et al., 2003) suggest that students’ perceptions of usefulness and ease of use shape their willingness to adopt mobile devices for learning purposes. However, from a cognitive load perspective (Sweller, 1988), the same devices may impose additional attentional demands, thereby increasing susceptibility to distraction.

Academic engagement, conceptualised as a multidimensional construct encompassing behavioral, emotional, and cognitive involvement (Fredricks et al., 2016), represents a proximal behavioral mechanism through which these cognitive evaluations are translated into learning-related actions. Importantly, engagement is not only an outcome but may also function as a regulatory process that shapes subsequent technology use patterns.

Drawing on these perspectives, we conceptualise a dynamic process in which students’ cognitive evaluations of mobile technology influence their behavioral regulation (i.e., engagement and distraction), which in turn feeds back into subsequent perceptions and usage patterns over time. This process-oriented view provides a theoretically grounded basis for examining reciprocal relationships among technology acceptance, digital distraction, and academic engagement in a longitudinal framework.

### Research gaps and theoretical integration

The dynamic interplay among technology acceptance, digital distraction, and academic engagement has not been comprehensively examined within a unified longitudinal framework. Three primary research gaps are identified. First, the predominance of cross-sectional designs (e.g., Liu et al., 2025) limits causal inference regarding the directional relationships among technology acceptance, digital distraction, and academic engagement, and cannot account for the possibility that highly engaged students may selectively adopt technology in more academically productive ways.

Second, most prior studies treat digital distraction as a unidirectional disruptor, without modelling the reciprocal feedback loop between cognitive appraisals, engagement states, and distraction levels (Ward et al., 2017).

Third, conventional cross-lagged panel models (CLPM) confound stable between-individual trait differences with within-individual dynamic processes, potentially biasing causal interpretations (Hamaker et al., 2015).

To address these gaps, the present study integrates the Technology Acceptance Model (Davis, 1989), Cognitive Load Theory (Sweller, 1988), Cognitive-Affective Systems Theory of personality (Mischel and Shoda, 1995), and the Triarchic Framework of Academic Engagement (Fredricks et al., 2016) to construct a theoretically integrated dynamic model.

This model conceptualises smartphone use in classroom settings as an iterative process involving cognitive evaluation of technology, behavioral regulation (engagement and distraction), and learning outcomes, in which contextual and individual boundary conditions shape the direction and magnitude of effects.

### Research objectives and hypotheses

The present study employs a three-wave longitudinal design (with six-month intervals) based on a sample of Chinese university students, and applies the Cross-Lagged Panel Model (CLPM; Hamaker et al., 2015).

The study has the following objectives:

(1) to investigate the reciprocal longitudinal relationships among technology acceptance, digital distraction, and academic engagement;
(2) to examine the empirical implications of mobile phone management policies in the Chinese educational context.

Based on the theoretical integration, the hypotheses of this study are as follows:

*H1a*: Technology acceptance at Time 1 positively predicts academic engagement at Time 2.

*H1b*: Technology acceptance at Time 1 positively predicts digital distraction at Time 2.

*H2a*: Digital distraction at Time 1 negatively predicts academic engagement at Time 2.

*H3a*: Academic engagement at Time 1 positively predicts technology acceptance at Time 2.

*H3b*: Academic engagement at Time 1 negatively predicts digital distraction at Time 2.

The hypothetical contribution of the study is the following: the paradigm of a tool/distraction dichotomy will be broken in order to demonstrate situational dependence and psychological dynamics of the educational role of mobile phones; methodologically, the study will develop longitudinal research paradigms in the field of educational psychology; and practically, differentiated interventions, i.e., increasing value-based guidance among people with cognitive biases toward technology and implementing metacognition training among easily distracted populations to help the mobile phones to change the status of a distraction into an educational facilitator (see the theoretical model). The results will help to have an empirical contextual ground in the implementation of the action plan of Education Informatization 2.0 in agreement with the more pronounced practice of the teaching-based on individual ability educational philosophy in the digital era.

## Research methods

### Study population

This study adopted a three-wave longitudinal design. The study population consisted of high school students from three public secondary schools (one each located in an urban, suburban, and rural area) in a province in eastern China. A stratified cluster sampling method was used: first, schools were stratified by location, and one school was randomly selected from each stratum; then, within each school, students were stratified by grade (Grades 10 through 12), and two classes were randomly selected from each stratum.

The initial sample consisted of 1,200 participants, with 1,020 successfully retained through the third wave (overall attrition rate: 15.0%). In the final analysis sample, there were 521 males (51.1%) and 499 females (48.9%); the mean age was 17.85 years (SD = 1.14); the grade distribution was as follows: 348 students in Grade 10 (34.1%), 362 students in Grade 11 (35.5%), and 310 students in Grade 12 (30.4%). All participants owned personal smartphones and carried them to school daily, meeting the requirements of the classroom smartphone-use context.

Data were collected in September 2023 (T1), March 2024 (T2), and September 2024 (T3), with a six-month interval between waves. The six-month interval was selected based on prior longitudinal research on technology-behavior relationships (e.g., Takahashi et al., 2026; Usami et al., 2019) and is consistent with natural academic semester boundaries, allowing sufficient time for meaningful behavioral change while maintaining ecological validity.

The data collection was approved by the Ethics Committee of Gansu Forestry Vocational Technical University (GSLYZYJSDX-20250912). Written informed consent was obtained from all participants and their legal guardians, including assurances of anonymity and the right to withdraw at any time.

Research assistants who had received standardized training administered group-based surveys in classrooms, and the average completion time for each questionnaire was approximately 25 min.

Little’s MCAR test (*χ*^2^ = 18.37, df = 24, *p* = 0.78) indicated that there were no significant differences between the dropout and retained groups in terms of gender, age, grade level, or baseline variables (all *p* > 0.05), suggesting that attrition was random and not systematic.

Follow-up analyses were conducted using full information maximum likelihood (FIML) estimation to handle missing data and to obtain unbiased parameter estimates (Enders, 2022).

### Research tools

#### Technology acceptance measure

Technology acceptance is defined as the degree to which an individual perceives that using a particular technology would enhance their performance and is willing to adopt it for that purpose (Davis, 1989; Venkatesh et al., 2012). It is conceptualised as a multidimensional construct encompassing both utilitarian and hedonic perceptions of technology use. The research number made use of the educational-context version of Unified Theory of Acceptance and Use of Technology (UTAUT2) scale by Venkatesh et al. (2012). The scale is based on 18 items and six dimensions that include performance expectancy (4 items), e.g., It additionally includes smartphone use, which proves my course content comprehension better (Using a smartphone helps me better understand course content) (3), effort expectancy (3), social influence (3), feasibility (3), hedonic motivation (3), and habit (2), items on the questionnaires. The scale uses the 7-point Likert scale (1 = strongly disagree, 7 = strongly agree), where the higher the score, the higher the technology acceptance. The initial scale had good psychometric effects in cross-cultural research (Chao, 2019). In this paper, the items were updated according to discussions that followed a panel of experts based on whether they were contextually appropriate in the environment of an educational set up, and whether it would result in a content validity. The 3 measurement waves of the Cronbach 3 coefficients of the total Technology Acceptance Scale was 0.91, 0.93, and 0.92; the 3 dimensions 2.2 coefficients were between 0.78 and 0.89. The results of the confirmatory factor analysis revealed that the six-factor model was of good fit (2/df = 2.83, CFI = 0.94, TLI = 0.93, RMSEA = 0.06), which also evidences that the scale was of good construct validity. This measurement has been shown to be of good application with the Chinese adolescents (Jin et al., 2025).

#### Classroom digital distraction scale

This study utilized the Classroom Digital Distraction Scale (CDDS) developed by Guo et al. (2024), which was specifically designed for the Chinese educational context. The scale comprises 15 items covering three dimensions: social media distraction (5 items, e.g., “I often cannot resist checking social media notifications during class”), gaming and entertainment distraction (5 items), and study-related digital interference (5 items). It employs a 6-point Likert scale (0 = Never, 5 = Always), with higher scores indicating more severe digital distraction. The scale’s design incorporated semi-structured interviews to ensure the cultural appropriateness of the content. The Cronbach’s *α* coefficients for the total scale across the three measurements in this study were 0.88, 0.89, and 0.90, respectively; the α coefficients for each dimension ranged from 0.79 to 0.87. A three-factor confirmatory factor analysis supported the scale’s structural validity (χ^2^/df = 3.12, CFI = 0.93, TLI = 0.91, RMSEA = 0.07). Correlation analyses between scale scores and self-reported mobile phone usage frequency (*r* = 0.67, *p* < 0.01) and ability to maintain attention in class (*r* = −0.58, *p* < 0.01) indicate good criterion-related validity. This tool has been validated for use with Chinese middle school students (Qiu et al., 2024).

#### Academic engagement scale

In this research, the Academic Engagement Scale including the revision by Xu et al. (2024) was used. This scale has three streams (dimensions) on the basis of the theoretical background of Fredricks et al. (2004), namely: behavioral engagement (5 items, e.g., I will continue to do academic work despite the challenges confronting me even though I will be measured using 14 items; even though 10 items will be in affective engagement (4 items) and cognitive engagement (5 items). It applies a Likert scale that consists of 5 items (1 strongly disagree, 5 strongly agree), and an increasing score means increasing levels of academic engagement. The instrument has been tested on a sample of Chinese adolescents and proves to have good psychometric characteristics. The internal consistencies represented by the Cronbachs *α* totals of the three measurements in the research were 0.87, 0.89 and 0.88 respectively; the Cronbachs 8 of each dimension in the research were between 0.76 and 0.85. The three-factor model was justified by the confirmatory factor analysis (2/df = 2.96, CFI = 0.95, TLI = 0.94, RMSEA = 0.05). The scale was highly related to academic performance (*r* = 0.43, *p* < 0.01), teacher-rated classroom participation (*r* = 0.51, *p* < 0.01), executing the criterion validity of the scale. This tool has been used in a variety of studies in Chinese educational research (Lei et al., 2018), which proves inter-sample consistency.

Discriminant validity was further assessed by examining the average variance extracted (AVE) for each construct. Results indicated that the square root of the AVE for each construct (technology acceptance: AVE = 0.62; digital distraction: AVE = 0.58; academic engagement: AVE = 0.55) was greater than its correlations with the other constructs, confirming adequate discriminant validity (Fornell and Larcker, 1981).

Additionally, the theoretical selection of subscale dimensions was guided by the UTAUT2 framework for technology acceptance (Venkatesh et al., 2012), the cognitive-behavioral framework for digital distraction (Guo et al., 2024), and the Triarchic Model of Academic Engagement (Fredricks et al., 2004, 2016), ensuring that each dimension corresponded to a theoretically meaningful component of the hypothesised mechanisms.

### Testing procedures and statistical analysis

All three testing sessions were conducted during regular class periods by research assistants who had undergone standardized training: before testing, the principles of anonymity and voluntariness were reiterated; procedural controls were implemented to reduce common-method bias (randomization of item order, inclusion of three attention check items, and use of reverse-scored items); instructions emphasized that responses should be based on “actual classroom experiences.” Each survey administration lasted approximately 25 min; questionnaires were collected on-site, and invalid responses were excluded based on predefined criteria (response time < 10 min, ≥2 errors on attention check items). Data were cross-checked by two researchers to ensure accuracy.

Data analysis was performed using SPSS 26.0 and Mplus 8.3. First, Harman’s one-factor test was conducted to assess common method bias, and descriptive statistics and Pearson correlation coefficients were calculated.

The core analysis involved the construction of a cross-lagged model. Model fit was assessed using χ^2^/df, CFI, TLI, RMSEA, and SRMR (Hu and Bentler, 1999 criteria: CFI/TLI > 0.90, RMSEA < 0.08, SRMR < 0.08). Full Information Maximum Likelihood (FIML) was used to handle random missing data, and model fit was compared using AIC, BIC, and the likelihood ratio test (Δ*χ*^2^). Three types of structural paths were estimated: (a) autoregressive paths, representing temporal stability; (b) cross-lagged paths (from one construct at an earlier time point to another construct at a subsequent time point), testing prospective predictive relationships; and (c) contemporaneous correlations among all variables at each time point, accounting for concurrent associations.

## Results

### Common method Bias testing

The present research used a two-fold approach of control procedures and statistical analyses to deal with the common method bias. Procedural level was administered in the form of anonymity, randomized presentation of items, use of reversed-scored items and attention-check items and separation of item order in independent and dependent variables (Podsakoff et al., 2003). At the statistical level: (1) Harman-single factor test (unrotated) the first factor explained 28.73% of variance (which is 40% of the variance) of the total of the items of the three waves of the core variables- all items were used in the analysis (47 items); (2) core item variance- 28.73 percent of the variable sample was too small to justify the rotation (Zhou Hao and Long Lirong, 2004). (2) Unmeasured Latent Method Factor (ULMC) strategy- a global method factor has been added to the structural equation model; The change in fit was 0.003 (<0.01) between the model with the method factor and the baseline model, which shows that the effect of the common method bias is negligible (Richardson et al., 2009). The outcome of both tests makes sure that the conclusion of this study is not highly influenced by common method biases and the quality of data is appropriate to the needs of the longitudinal analysis.

Descriptive statistics indicate that the mean score for technology acceptance exhibited a slight upward trend (ΔM = 0.16), the level of digital distraction increased gradually (ΔM = 0.17), and academic engagement also showed a modest increase (ΔM = 0.16). Standard deviations decreased over time, indicating that within-group variability was converging. Correlation analysis revealed that (See Table 1): (1) significant autocorrelations were observed across time for all variables (*r* = 0.48–0.67, *p* < 0.01), which confirms the temporal stability of the constructs; (2) at each time point, technology acceptance was positively correlated with digital distraction (*r* = 0.17–0.27, *p* < 0.01) and also positively correlated with academic engagement (*r* = 0.21–0.31, *p* < 0.01); (3) digital distraction and academic engagement were significantly negatively correlated at all time points (*r* = −0.31 to −0.41, *p* < 0.01). These patterns provide preliminary support for the underlying causal pathway of “technology use—distraction—academic engagement” and lay an empirical foundation for subsequent cross-lagged models to examine the longitudinal predictive directions and mediating mechanisms among the variables.

**Table 1:** Descriptive statistics and correlation coefficients for each variable.

Variable	*M*	*SD*	1	2	3	4	5	6	7	8	9
1. TA T1	4.52	1.18	—								
2. TA T2	4.61	1.09	0.63	—							
3. TA T3	4.68	1.03	0.59	0.67	—						
4. DD T1	3.18	1.27	0.24	0.19	0.17	—					
5. DD T2	3.26	1.19	0.21	0.27	0.20	0.53	—				
6. DD T3	3.35	1.12	0.18	0.22	0.25	0.48	0.56	—			
7. AE T1	4.03	0.98	0.29	0.24	0.21	−0.39	−0.34	−0.31	—		
8. AE T2	4.11	0.91	0.27	0.31	0.25	−0.37	−0.41	−0.35	0.60	—	
9. AE T3	4.19	0.89	0.24	0.28	0.30	−0.34	−0.38	−0.40	0.57	0.63	—

TA, Technology acceptance; DD, digital distraction; AE, academic engagement were measured at three time points (T1, T2, T3).

### Cross-lagged analysis of technology acceptance and academic engagement

As shown in Table 2 and Figure 1, the analysis based on the conventional cross-lagged panel model (CLPM) indicates that the model fits well (CFI = 0.95, RMSEA = 0.06), supporting the adequacy of the theoretical model. All autoregressive paths were of great essence (= 0.58 0.65, p = Acceptance of technology and academic engagement had high temporal stability). Some of the significant cross-lagged predictive relationships included T1 in T2 academic engagement: although the predictive effect of academic engagement on future technology acceptance was significant (0.16), it reduced over time (T1 in T2: 0.16 0.11), and T2 in T3 (0.19) academic engagement: the predictive effect of technology acceptance on future academic engagement was smaller (0.11), and the trend was downward (T2 in T3: 0.19). The comparison of path coefficients reveals that the effect size of the Academic Engagement to Technology Acceptance (0.175) was much higher on the positive side compared to the reverse path (0.10), as it could represent the experiences that the students had in the learning process as the active antecedent of forming the perception of the value of classroom technology tools. To some extent, these findings confirm the fundamental process of technology acceptance theory that support the use experience reinforces the perceived usefulness (Davis, 1989) and also translate the results that, in classrooms, the state of academic involvement may have a more predominant role in attitudes, to technology, than direct effect of technological tools on academic task performance. This result can be taken as preliminary longitudinal evidence of the dynamism of the role of the mobile phone: at the point of strong engagement of the students in the learning process, they tend to consider mobile phones as useful tool; nevertheless, the low impact of technology acceptance on academic activities is also a reminder that the willingness to use technology needs to be reinforced by the protection against the potential distracting tendency and possible ways. This discussion forms the empirical basis of further integrated three-variable models including digital distraction. All fit indices met or exceeded the recommended thresholds (Hu and Bentler, 1999: CFI/TLI >0.90, RMSEA <0.08).

**Table 2:** Path coefficients of the cross-lagged model of technology acceptance and academic engagement.

Path	β	*SE*	*p*
Autoregressive paths			
TA T1 → TA T2	0.62	0.03	<0.001
TA T2 → TA T3	0.65	0.03	<0.001
AE T1 → AE T2	0.58	0.04	<0.001
AE T2 → AE T3	0.61	0.04	<0.001
Cross-lagged paths			
TA T1 → AE T2	0.11	0.04	0.007
AE T1 → TA T2	0.16	0.04	<0.001
TA T2 → AE T3	0.09	0.04	0.021
AE T2 → TA T3	0.19	0.04	<0.001
Model fit indices	χ^2^/df = 2.69, CFI = 0.95, TLI = 0.93, RMSEA = 0.06 [0.04, 0.08]		

**Figure 1:**
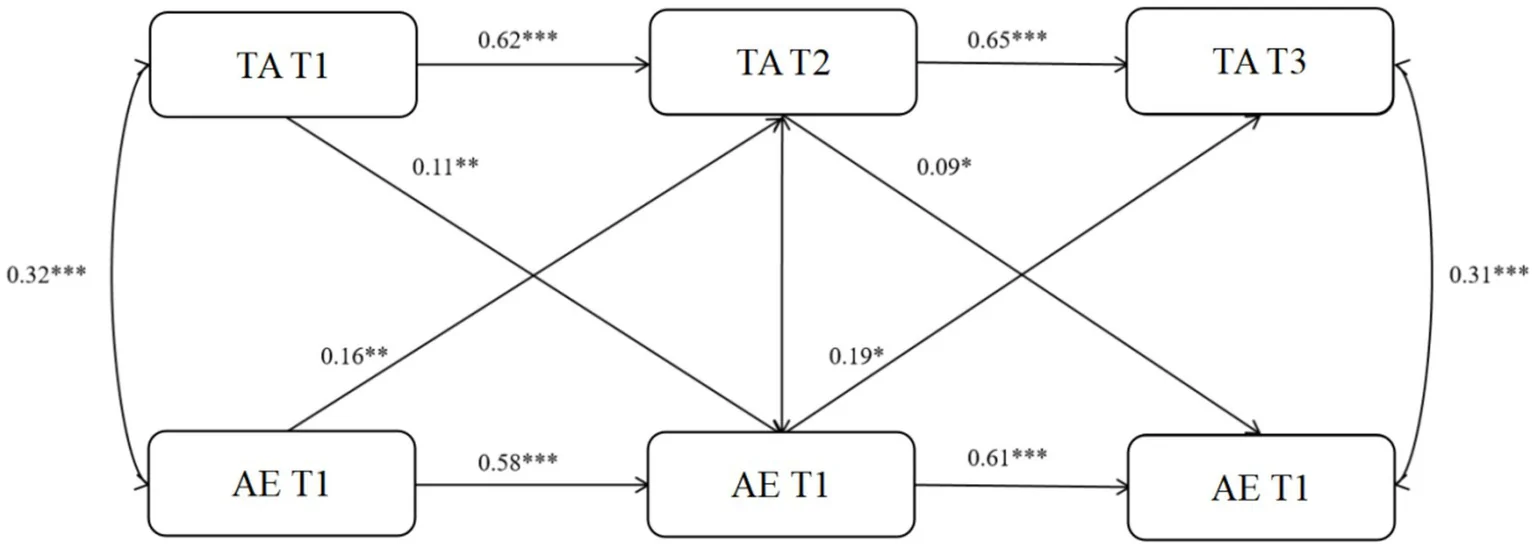
Cross-lagged model of technology acceptance and academic engagement.

Cross-lagged paths revealed complex longitudinal relationships among variables, as shown in Table 3 and Figure 2. First, technology acceptance significantly and positively predicted subsequent digital distraction (*β*T1 → T2 = 0.14, *p* < 0.001; βT2 → T3 = 0.12, *p* < 0.01), but its direct effect on subsequent academic engagement was not significant. Second, digital distraction significantly and negatively predicted subsequent academic engagement (βT1 → T2 = −0.23, *p* < 0.001; βT2 → T3 = −0.25, *p* < 0.001). Third, academic engagement not only negatively predicted subsequent digital distraction (βT1 → T2 = −0.08, *p* < 0.05; βT2 → T3 = −0.09, *p* < 0.05), but also positively predicted subsequent technology acceptance (βT1 → T2 = 0.18, *p* < 0.001; βT2 → T3 = 0.20, *p* < 0.001). Further longitudinal mediation analysis (5,000 bootstrap resamples) indicated, as shown in Table 4, that digital distraction fully mediated the relationship between technology acceptance and academic engagement. Specifically, the indirect effect of T1 technology acceptance on T3 academic engagement through increased T2 digital distraction was significant (*β* = −0.032, 95% CI = [−0.059, −0.013]), whereas the direct effect of T1 technology acceptance on academic engagement was not significant (*β* = 0.016, 95% CI = [−0.055, 0.086]).

**Table 3:** Path coefficients for the three-variable cross-lagged model.

Path	T1 → T2 β (SE)	T2 → T3 β (SE)
Autoregressive paths		
TA → TA	0.64 (0.03)***	0.66 (0.03)***
DD → DD	0.53 (0.04)***	0.56 (0.04)***
AE → AE	0.59 (0.04)***	0.62 (0.04)***
Cross-lagged paths		
TA → DD	0.14 (0.04)***	0.12 (0.04)**
TA → AE	0.05 (0.04)	0.04 (0.04)
DD → AE	−0.23 (0.04)***	−0.25 (0.04)***
DD → TA	0.02 (0.04)	0.01 (0.04)
AE → TA	0.18 (0.04)***	0.20 (0.04)***
AE → DD	−0.08 (0.04)*	−0.09 (0.04)*

All models controlled for time-varying residuals. *p < 0.05, **p < 0.01, ***p < 0.001.

**Figure 2:**
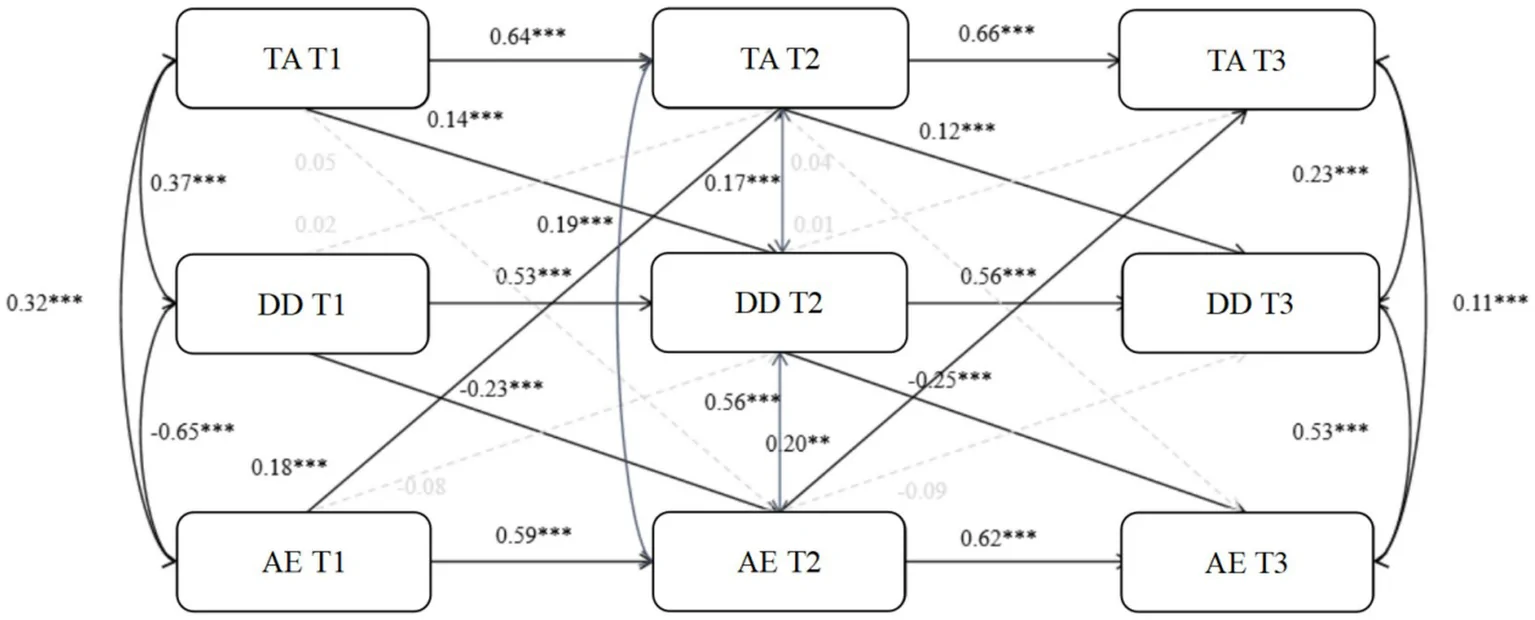
Path diagram of a three-variable cross-lagged model.

**Table 4:** Test of the longitudinal mediation effect of digital distraction (5,000 bootstrap repetitions).

Path	Effect Value	95% CI
T1 TA → T2 DD → T3 AE	−0.032	[−0.059, −0.012]
T1 TA → T3 AE (Direct Effect)	0.018	[−0.052, 0.087]
Total effect	−0.016	[−0.055, −0.005]

The non-significant direct effect of technology acceptance on academic engagement (*β* = 0.016, 95% CI = [−0.055, 0.086]) is theoretically meaningful and warrants further interpretation. This finding suggests that technology acceptance does not directly enhance or impair academic engagement; rather, its influence is fully mediated by digital distraction. In other words, students’ positive perceptions of smartphone usefulness translate into increased in-class phone use, which in turn elevates distraction levels that subsequently undermine engagement.

This pattern of full mediation is consistent with cognitive load theory (Sweller, 1988), which posits that the cognitive resources consumed by digital distraction reduce the capacity available for learning-related processing, thereby offsetting any potential benefits associated with technology acceptance and resulting in no direct effect on academic engagement.

Following Cohen’s (1988) guidelines for interpreting effect sizes in behavioral research (*β* = 0.10 small, 0.30 medium, 0.50 large), the cross-lagged effects observed in this study ranged from small to medium in magnitude. Specifically, the effect of technology acceptance on digital distraction (*β* = 0.12–0.14) was small, whereas the effect of digital distraction on academic engagement (*β* = −0.23 to −0.25) approached the threshold of a medium effect size, indicating a practically meaningful negative impact. The effect of academic engagement on technology acceptance (*β* = 0.18–0.20) fell within the small-to-medium range.

These magnitudes are consistent with those reported in comparable longitudinal studies examining technology–behavior relationships (e.g., Takahashi et al., 2026; Núñez-Regueiro et al., 2022).

### Summary of hypothesis testing results

Table 4 summarises the results of hypothesis testing across the two cross-lagged intervals. Of the six hypothesised cross-lagged relationships, five were empirically supported. H1a (technology acceptance → academic engagement) was not supported, as the direct effect was non-significant in both intervals. H1b (technology acceptance → digital distraction) received support, indicating that higher technology acceptance predicted increased subsequent digital distraction.

H2a (digital distraction → academic engagement) was supported and exhibited the strongest effects among all cross-lagged paths, confirming that digital distraction is a robust negative predictor of academic engagement. H3a (academic engagement → technology acceptance) was supported, demonstrating that academically engaged students develop more positive technology perceptions over time. H3b (academic engagement → digital distraction) was supported, indicating that engaged students are better able to resist digital distractions.

The non-significant H1a result, together with the significant indirect effect via digital distraction, provides evidence for the full mediation model proposed in this study.

## Discussion

Drawing on three-wave longitudinal data and cross-lagged panel modelling, this study examined the reciprocal temporal relationships among technology acceptance, digital distraction, and academic engagement in Chinese high school students. The findings provide longitudinal evidence challenging the simplistic “tool vs. distractor” binary and instead reveal context-dependent and dynamic mechanisms through which mobile phone use shapes educational outcomes. The discussion that follows addresses four interrelated themes: theoretical integration, mechanistic explanation, methodological contributions, and practical implications.

### The nonlinear relationship between technology acceptance and learning outcomes: from tool value to behavioral cost

This research obtained that the direct predictive value of technology acceptance on educational participation was frail and unsteady and it did not entirely concur with the guess of the customary Technology Acceptance Model (TAM) (Davis, 1989). This observation lies in one of the fundamental principles of cognitive load theory, according to which the educational usefulness of technological tools can not just be defined by the perceived value of the tools but is also limited by the fact that the limited cognitive resources of learners are distributed (Sweller et al., 2019). In case the students put higher importance on the learning advantages of smartphones (high technology acceptance) they can in fact use the smartphones more often in classes, which will catalyze the digital distraction and ultimately counter the possible advantages of the technology. The given effect of paradox was also preliminately confirmed in the research by Qi (2019), who discovered that although academic utilization of mobile devices by students boosted the efficiency of information retrieval, it also increased the level of technostress and deteriorated academic performance at the same time. The longitudinal design was used in this study, further supporting that academic engagement is mainly mediated by a complete mediation strategy that incorporates digital distraction because technology acceptance has a two-sided concept to technology-integrated learning spaces.

It is interesting to note that the predictive relationship between academic engagement and technology acceptance was quite strong in contrast to the reverse relationship. Such an outcome confirms the cognitive-affective system theory as proposed by Mischel and Shoda (1995) where the behavior of an individual experienced in particular situations transforms his/her cognitive judgment about environmental stimuli. Highly academic students accrue effective learning experiences throughout the learning process; these effective learning experiences are transferred to their assessment of learning tools, requiring them to be more likely to perceive the learning utility of smartphones. This conclusion can be drawn based on the results of Jin et al. (2025): concerned students can recognize the pedagogical potential of new technologies like generative AI better when they are intensely involved in learning activities. It indicates that maximizing the academic engagement of the students can be an effective approach to maximizing the adaptability of the educational technology, instead of the unidirectional trajectory that is conventionally suggested, that is, to increase the ability to accept technological innovation and, subsequently, to maximize the academic engagement.

### The bidirectional mechanism of digital distraction: the interaction between situational perception and self-regulation

This experiment indicated the digital distraction was not only one of the undesirable effects of using technologies but also one of the main mediating variables between cognitive appraisal and learning behaviors. Prior to subsequent digital distraction, technology acceptance significantly and positively predicted the evidence. Although this outcome may appear paradoxical, such a finding, in fact, disclosed the multifaceted interaction between the perception of technology and the behavioral practice. Through the students, who have a high conviction in the learning potential of the smartphone, there is a high chance that the phone can be used more in class, but without effective self-regulation then the use of smartphones can be more inclined to non educational purposes (Ophir et al., 2009). This result was consistent with the theory of brain drain by Ward et al. (2017): even having self-motivation to use mobile phones to facilitate learning, only the very existence of the gadget devotes finite resources to cognition and decreases the ability to process essential knowledge meaningfully.

In contrast, the high level of prediction against digital distraction on academic engagement substantiated the principle of attentional resources limitation. The constant change of attention brought about by the smartphones (average once every 90 s) makes it hard to enter a state of deep concentration among students leading to an interrupted learning process (Levy et al., 2012). Nevertheless, this paper also discovered that academic absorption played an important role in impugning future online distraction, showing that readily engaged learners had a better self-directing capacity and were in a position to withhold irrelevant stimulation when required. This result corresponded to the position of Bylieva et al. (2021) in the importance of self-regulation in e-learning settings and to the view of Fredricks et al. (2016) the tripartite model of academic engagement according to which behavioral, affective, and cognitive engagements are combined to create a psychological defense against distractions.

The most interesting was the complete mediation effect of digital distraction between the technology acceptance and academic engagement; this result was contrary to the idea that there was a direct relationship between the technology characteristics and learning outcomes as found in traditional educational technology studies. The technological tools did not have an intrinsic educational value but rather constructed dynamically both in the behavior practice of users as well as the cognitive experience. The adoption of technology when it becomes uncontrolled in the use will result in learning impairment even though the adoption may result in an involvement of learning since it is distracting and disruptive with regard to technology. Such a dynamic transformation mechanism can be used to describe the situation when the same technological tool could have significantly different impacts on different students or in various contexts (Zou et al., 2025).

### Research limitations and future directions

This study has several limitations that should be acknowledged. First, the sample was restricted to high school students in a single province in eastern China, which limits the generalisability of the findings to other cultural and educational contexts. Future research should replicate this model using more diverse regional and cross-cultural samples to examine boundary conditions (Guo et al., 2024).

Second, all variables were measured using self-report scales, which may introduce common method variance. Although procedural controls and the Unmeasured Latent Method Factor (ULMC) approach were applied, future studies should incorporate objective behavioral measures (e.g., device-level screen time logs and eye-tracking data) to improve validity (Qiu et al., 2024).

Third, the conventional cross-lagged panel model (CLPM) used in this study does not separate stable between-person differences from within-person dynamics (Hamaker et al., 2015). Future research should adopt the random-intercept cross-lagged panel model (RI-CLPM; Mulder and Hamaker, 2021) to obtain more precise within-person estimates and to determine whether the observed relationships reflect true intraindividual processes or stable trait-like differences.

Fourth, the six-month interval between waves may not capture short-term fluctuations in technology use and distraction. Future studies could employ experience sampling methods (ESM) to capture fine-grained temporal dynamics (Usami et al., 2019).

Finally, future research may extend this work in three directions: (1) examining individual differences such as self-control and metacognitive strategies; (2) exploring contextual boundary conditions such as classroom management and instructional design; and (3) adopting mixed-method or cognitive neuroscience approaches (e.g., fNIRS, EEG) to investigate the neurocognitive mechanisms underlying digital distraction (Shanmugasundaram and Tamilarasu, 2023).

### Implications for practice

The findings of this study provide empirical evidence for educational policymakers and challenge the binary view of “total prohibition” versus “complete openness.” First, simply increasing technology adoption without accompanying strategies to manage distractions may lead to negative outcomes. This suggests that schools must simultaneously strengthen digital literacy and self-regulation training when implementing “BYOD” (Bring Your Own Device) policies. The “phone-away strategies” (e.g., placing phones out of sight) suggested by Ward et al. (2017) may serve as a low-cost intervention.

Second, enhancing academic engagement may be a prerequisite for optimizing technology use. Teachers can boost student motivation by designing highly engaging learning tasks (such as problem-based learning and collaborative inquiry), thereby fostering students’ ability to use technology critically (Xu et al., 2024). This “inside-out” strategy aligns more closely with constructivist learning principles than simply restricting phone use.

Third, differentiated strategies should be adopted for different student groups. This study found significant individual differences in the effects of technology use; students with high self-control may benefit from using smartphones in class, while those with low self-control face a greater risk of distraction. Schools can establish “technology use agreements” that allow students to choose different usage strategies based on self-assessment, reflecting the principles of personalized education (Şahin and Yıldız, 2024).

Finally, teacher professional development should be integrated into digital distraction management strategies. Research by Huang and Teo (2020) indicates that teachers’ acceptance of technology and confidence in its use directly influence students’ patterns of technology use. By training teachers to design “distraction-proof” classroom structures—such as clearly defining technology usage periods, tasks, and objectives—smartphones can be transformed from potential sources of disruption into structured learning tools (Jin et al., 2025).

## Conclusion

This research demonstrates that the role of mobile phones in learning environments is neither a fixed learning-enhancing tool nor an unavoidable source of distraction, but rather a dynamic factor shaped by technology-related cognition, behavioral control, and learning context.

To capture the educational significance of technology adoption, this study suggests that a transformative process linking cognitive appraisal, behavioral regulation, and learning outcomes occurs during technology use, in which digital distraction serves as an important gatekeeping mechanism.

Accordingly, mobile phones can enhance learning only when students possess sufficient self-regulation skills, clearly defined learning goals, and appropriate contextual support.

It is recommended that educational technology design be guided by an integrated framework combining embodied cognition theory and cognitive load theory, emphasising dynamic and context-sensitive technology use.

Future policies on educational digitalisation should move beyond simply measuring device penetration rates and instead focus on developing students’ technology-related self-regulation skills and critical digital literacy, thereby supporting evidence-based digital literacy interventions grounded in the empirical findings of this study.

## Data Availability

The raw data supporting the conclusions of this article will be made available by the authors, without undue reservation.
